# Tipping Bucket Rain Gauges in Hydrological Research: Summary on Measurement Uncertainties, Calibration, and Error Reduction Strategies

**DOI:** 10.3390/s23125385

**Published:** 2023-06-07

**Authors:** Daniel A. Segovia-Cardozo, Carlota Bernal-Basurco, Leonor Rodríguez-Sinobas

**Affiliations:** Research Group Hydraulics for Irrigation, Departmento de Ingeniería Agroforestal, Escuela Técnica Superior de Ingeniería Agronómica, Alimentaria y Biosistemas, Universidad Politécnica de Madrid, Ciudad Universitaria s/n, 28040 Madrid, Spain

**Keywords:** precipitation monitoring, rainfall measurement biases, rain gauge, measurement error, hydrological forecast

## Abstract

Tipping bucket rain gauges (TBRs) continue to be one of the most widely used pieces of equipment for rainfall monitoring; they are frequently used for the calibration, validation, and downscaling of radar and remote sensing data, due to their major advantages—low cost, simplicity and low-energy consumption. Thus, many works have focused and continue to focus on their main disadvantage—measurement biases (mainly in wind and mechanical underestimations). However, despite arduous scientific effort, calibration methodologies are not frequently implemented by monitoring networks’ operators or data users, propagating bias in databases and in the different applications of such data, causing uncertainty in the modeling, management, and forecasting in hydrological research, mainly due to a lack of knowledge. Within this context, this work presents a review of the scientific advances in TBR measurement uncertainties, calibration, and error reduction strategies from a hydrological point of view, by describing different rainfall monitoring techniques, summarizing TBR measurement uncertainties, focusing on calibration and error reduction strategies, discussing the state of the art and providing future perspectives of the technology.

## 1. Introduction

Rainfall is a phenomenon that has attracted the interest of human beings since ancient times; we have tried to measure it and to understand all the processes linked to it. The earliest reference to a rain gauge was in India, by Kautilya, in the fourth century B.C.; however, references regarding its use in Europe started in the 17th century in Italy, where Benedetto Castelli developed isolated experiments with a non-recording rain gauge in around 1639 and Sir Christopher Wren developed the earliest recording instrument [1]. Since then, this interest has grown, and nowadays, rainfall monitoring, research, and forecasting (calibration/validation) are of general interest to better understand the hydrological cycle and subjects that depend on water and precipitation, such as irrigation, hydrological research, water resource management, flood risk, flood alert, flood and weather forecast, hydraulic structure design, urban planning, industry, and a large number of human interest activities [2,3,4,5,6,7,8,9,10]. Human interest in precipitation monitoring has grown with technological development and the increase in human population, which demands increasingly more resources, generating stress on natural hydrological systems. Thus, hydrological research is gaining interest not only in the scientific community, but also in governmental institutions, with unprecedented support [11]. However, hydrologic systems are complex and to better address hydrological research, modeling, and forecasting, accurate data, and if possible, data obtained with a high space–time frequency are needed to understand and explain the nature of dynamical hydrologic systems and to help with decision making aimed at mitigating flood risk, among other branches of hydrological research [11,12,13,14,15,16].

Moreover, the goals of the current International Hydrologic Scientific Decade 2013–2022 highlight these challenges, and call for the development of new and innovative monitoring techniques to improve hydrologic monitoring platforms, networks, and databases in order to obtain more reliable data [12,15].

Among the different hydrological variables, rainfall monitoring is one of the most important factors in hydrological research and flood risk management [11,17]. Monitoring rainfall in time and space, together with the availability of accurate rainfall data, is a key issue for managing water resources and forecasting, such as water budget studies, flood frequency analysis, sewer drainage design, surface water hydrology, and flood risk [16,18].

Accurate rainfall data are needed to understand the complexity of hydrological systems for water management and rainfall flood forecast, and to better understand extreme events, which can cause severe damage; these are scarcely documented and lead to difficulties in flood modeling [19,20]. Since precipitation (depth and intensity) is usually estimated by means of temporal averages and spatial interpolations that often do not represent their variability, accurate and reliable results can be achieved if good-quality hydrologic data are provided from a well-designed and well-implemented data collection program [11,21].

Measuring rainfall is not only about instrument accuracy; it is also about the capacity of the data to represent the reality of the hydrological system. Since the rainfall phenomenon is characterized as being dynamic, not only in time but also in space, a proper frequency of data measurement and its spatial representativeness must be considered to achieve better results. As [22] commented, the starting point in gaining a good hydrological understanding is accurate rainfall measurement and forecasting with useful lead times. Nonetheless, instrument accuracy is a main factor to be considered, since most of the instruments used to measure rainfall are subject to measurement biases that can be reduced with proper equipment management [2].

## 2. Rainfall Measurement

Water is a key resource for human societies, a resource that falls from the sky and generates a multitude of essential processes, but sometimes it is also detrimental to the development of human activities. The measurement of precipitation is of interest to humans and is a global concern due to the increment in water stress, promoting the development of several instruments and techniques for its measurement and estimation, such as different rain gauges or the use of remote sensing, constantly being improved to reduce bias and catch the space and time variability in rainfall.

Rainfall measurement techniques can be classified into two categories: ground-based monitoring (field sensors) and space-based measurement (remote sensing). However, at the moment, space-based measurements require ground-based data for calibration and validation purposes [18].

### 2.1. Ground–Based Rain Gauges

According to [8], “rain gauge” is a broad term that refers to any instrument used to measure the amount of liquid precipitation over a set period. There are two different kinds of rain gauges: point rain gauges and extended ground remote measurement sensors such as radar, which are most frequently used for rainfall estimation at catchment scales [8].

#### 2.1.1. Point Rain Gauges: Catching and Non-Catching Point Rain Gauges

Point rain gauges measure the amount of precipitation at a point location, over a set period of time, mainly by direct or indirect rainfall collection [8]. They are the most widely used rainfall sensors and provide good accurate measurements near the ground surface and at a low cost compared to other sensors [8,23,24]. Likewise, their records are often considered as real ground measurements in radar and remote sensing data calibration and verification [23,24]. Considering the way they measure precipitation, two types of point rain gauges are distinguished: catching rain gauges, which directly measure precipitation through different mechanisms, and non-catching rain gauges, which infer precipitation through its relationship with other variables.

Catching point rain gauges measure the amount of precipitation at a point location, over a set period, mainly by means of direct rainfall collection. These are the oldest and most common sensors in field monitoring and can be further categorized into non-recording and recording gauges. It is important to state that catching rain gauge records (especially their effective temporal resolution and accuracy) are influenced by the nature of their mechanisms, which can define their measurement time interval [8,25].

Non-recording point rain gauges collect rainfall volumes over a given period, which need to be measured manually by an operator, while recording gauges provide automatic records of rainfall by mechanical and/or electronic mechanisms; they can provide rainfall estimates at a finer temporal resolution (usually related to the precision of the instrument and rainfall intensity) compared to non-recording gauges. Tipping bucket, weighing, and level gauges are some examples of these gauges [8,25,26].

Recording gauges are the preferred choice for rainfall monitoring at the catchment scale, while non-recording gauges are frequently used as a reference for quality control of the first [8]. The most common types of automatic recording gauges are TBRs [8,27]. However, weighing gauges offer a similar performance to TBRs and are becoming popular due to their lower maintenance requirements [8,25].

On the contrary, non-catching point rain gauges measure the amount of precipitation, at a point location over a set period of time, through different parameters (e.g., optical, microwave, acoustic, and piezoelectric disdrometers) [8]. They do not need a funnel to collect precipitation, since they detect individual hydrometeors as they pass through the measurement area or impact the sensor. They are known as disdrometers, due to their capacity to provide additional information, such as hydrometeor size distribution and fall velocity, further than precipitation accumulation or intensity. In addition, their low-maintenance cost has increased their application in recent years [28]. Nevertheless, the performance of disdrometers is lower than that of traditional rain gauges, since they lack standardized calibration and recognized testing methods in the laboratory [29,30].

#### 2.1.2. Radar

Radars measure rainfall indirectly by sending pulses of electromagnetic radiation into the atmosphere and recording the return signals; they are used to estimate rainfall rates at a given distance from the radar, since the quantity of reflected energy is a function of particle size, type, and distribution. The radar’s effective spatial–temporal resolution (generally of the order of, and often coarser than, 1 km^2^ and 1 h for radar rain gauge merging) and accuracy are determined by radar hardware, scanning strategy, distance from the radar, atmospheric conditions, and rainfall estimation algorithms [8,31,32].

Rain gauges deliver relatively accurate point rainfall estimates; nonetheless, they cannot capture the spatial variability of rainfall, [8,33,34]. In contrast, radars can better capture the spatial variability of rainfall. However, according to [8], the accuracy of radar measurements is generally insufficient for extreme rainfall events, it “is virtually impossible to have error-free radar quantitative precipitation estimates, and the new radar technologies and processing techniques will not change the inherent limitations of radar”. TBRs are the main data source used for the adjustment and validation of radar rainfall estimates [7,8,35,36].

Weather radars focus on polarized electromagnetic radiation pulses of a given frequency (*f*) and wavelength (*λ*) into a beam of angular width *θ* through a parabolic antenna. Each pulse travels at the speed of light and is backscattered by precipitation [8,32]. According to *f* and *λ*, weather radars can be classified into three main types (from higher to lower *f*): X-band, C-band, and S-band. The higher the frequency, the shorter the wavelength; therefore, high-frequency radars can detect smaller particles, and they are smaller and cheaper but also less powerful than low-frequency ones, which results in a shorter radar range and higher susceptibility to attenuation by strong rainfall intensities. 

S-band radars are frequently used to monitor severe weather phenomena in long ranges, such as tropical storms [8]. C-band radars provide a good compromise between cost and power, being the most widely used in Europe [8,37]. X-band radars are the smallest, most portable, and cheapest of all and are commonly used to study cloud development and monitor rainfall over small areas, such as urban areas, where their use has been gaining interest in recent years [8,38,39,40,41,42].

### 2.2. Satellite Remote Sensing

Although ground-based radar technology, described in the previous section, is considered a remote sensing technology, in this section, it refers to satellite remote sensing, which also includes satellite radar sensors.

In the last two decades, the use of remote sensing in hydrological research and monitoring, especially in flood forecasting, has increased considerably. Remote sensing of products is commonly used to estimate precipitation, soil moisture, elevation data, and snow cover. Precipitation remote products are used to overload the gauged rainfall data gap (e.g., ungauged areas) to improve hydrological management and forecasts [43]. 

Infrared and microwave technologies have been the most widely used for satellite precipitation monitoring since the 1970s and 1980s, respectively [43,44]. Precipitation is estimated, through different algorithms, on the quantitative relationship between cloud-top temperature and rainfall rate, such as the PERSIANN-CCS [45] and NRL-Blend high resolution [46], as well as others [45,47,48,49,50]. Remote sensing techniques can provide spatially distributed rainfall estimates to cover the lack of data in ungauged watersheds; however, there is no guarantee that reliable simulations can be generated if compared with gauged rainfall records [43,51].

At present, remote sensing has some limitations, such as low spatial–temporal resolution, especially in free products, and requires ground-based sensors for calibration and downscaling; which increases the errors in rainfall estimation (overestimation) [52,53,54]. Barrett and Beaumont [55] affirmed that “the problems facing satellite rainfall researchers are considerable, and even in some respects virtually insuperable”, and to date, they have not been completely overcome.

### 2.3. Other Indirect Measurement Systems

In addition to the use of satellites, radars, and weather stations, other sources of precipitation data have come about at the hand of the Internet of Things (IoT), big data, and 5G networks for rainfall monitoring [56]. Thus, the use of unmanned aerial vehicles and smartphone technologies, such us the existing physical measurements in wireless microwave signals, as the commercial cellular communication networks for near-ground rainfall monitoring, has increased [56,57,58,59]. Some examples of the latest microwave technology include E-band (links, multi-band boosters, and line-of-sight multiple-input multiple-output (LOS-MIMO) backhaul links) [59]. 

Cellular microwave backhaul links, densely deployed around the world, have the potential to complement precipitation monitoring systems, especially in urban areas, but also remote and complex topography areas, where traditional monitoring equipment cannot be easily installed [56,58]. They can accurately measure near-ground rain rates with a high time resolution, and offer a promising technology in rainfall monitoring for hydrological research and forecasting, which could assist in flash flood warning systems [59,60,61].

Vertical cloud or 3D cloud structure reflectivity is another interesting technology that can monitor and forecast precipitation, based on the analysis of cloud structures to estimate precipitation through cloud droplet distribution, liquid water content, cloud water paths, etc. However, current techniques are limited and cannot detect large-scale 3D cloud structures, limiting their ability to understand and forecast clouds and precipitation, since their spatial coverage is very limited and their temporal resolution is low. Vertical cloud structures can be obtained by soundings, laser, aircrafts, radars, and satellite sensors [62]. 

## 3. Tipping Bucket Rain Gauges and Their Measurement Uncertainties

**Figure 1:**
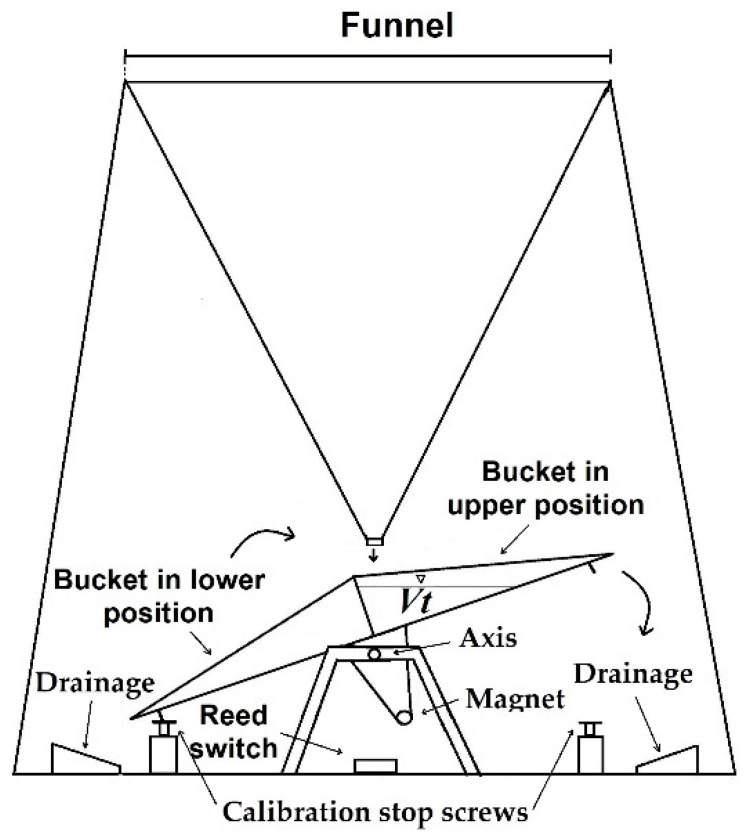
Tipping bucket rain gauge.

Among all the different rainfall measurement sensors, TBRs are the most widely used worldwide due to their simple manufacturing structure, which is based on an electric pulse, a magnetic reed switch mechanism, and a counter [2]. TBRs include a receiver (a funnel) that collects rainwater into a bucket, which is one-half of a two-bucket receptacle, pivoted on a cylindrical axis, that tips once the upper bucket fills up to a specified water amount (tipping volume, *Vt*) and raises the lower bucket into a position under the waterfall line, where it will begin to fill at the same time as the other bucket empties. In this movement, a magnet located in the tipping bucket mechanism generates an output signal through a magnetic reed switch to a recorder, and each pulse is set as a nominal amount of rainfall according to a fixed *Vt*, which can be set by two calibration stop screws (one for each bucket; Figure 1) [2,28,63].

TBRs have become one of the most extensive rain gauges, due to their simple manufacturing structure, low cost of production, and energy-saving capacity (a key element for the manufacturing of most weather instruments), being commonly used by National Meteorological Services, airports, industries, farmers, and private individuals to measure the rain depth and intensity [2,28,64]. In hydrological research, they are the most common gauges used to measure precipitation, which is one of the main variables for runoff models, calibration of distributed precipitation measurements [3,4,65], downscaling of remote sensing precipitation models [6,66], radar calibration [7,40,67], basin water balance, flood models [23], structure design, and profitability in different projects (hydroelectrical generation, dams, irrigation, city planning, etc.) [2,9].

Despite the advantages of TBRs, their main drawbacks are due to measurement errors and uncertainties originating in the operation of the equipment itself, as well as by external agents, which impacts on measurement reliability. Numerous studies in recent years have focused on explaining the nature of these measurement biases and have proposed different calibration methodologies and strategies to reduce the measurement uncertainty in TBRs, and some book chapters have addressed this topic [30,68]. However, to the best of our knowledge, there are no review articles on TBR measurement uncertainties, calibration, or error reduction strategies. This is the main objective of this work, with the purpose of gathering the knowledge developed in the area and making it more accessible.

According to [68], all instruments have biases and uncertainties, but in precipitation, their quantification and adjustment are more complex than for other environmental variables due to the variability of hydrometeors. However, this aspect is often forgotten by managers of operational monitoring networks and data users; consequently, biases are forwarded through applications or modeling, resulting in a lack of reliability [65,69,70]. These biases are usually considered by correction models that modify the measured precipitation, adding biases generated by wind, wetting, evaporation, and mechanical biases [31].

TBR rain measurements are affected by systematic biases due to instrumental, environmental, and management sources. In general, an underestimation of total rainfall can be observed, due to the difficulty of TBRs to catch the exact amount of water falling over the projection of the measurement area on the ground (wind, evaporation, splashing, and wetting phenomena) and to correctly measure the amount of water collected by its funnel (mechanical biases). In TBRs, the main sources of measurement biases are those caused by wind and mechanical biases [68,71].

### 3.1. Instrumental Error

**Figure 2:**
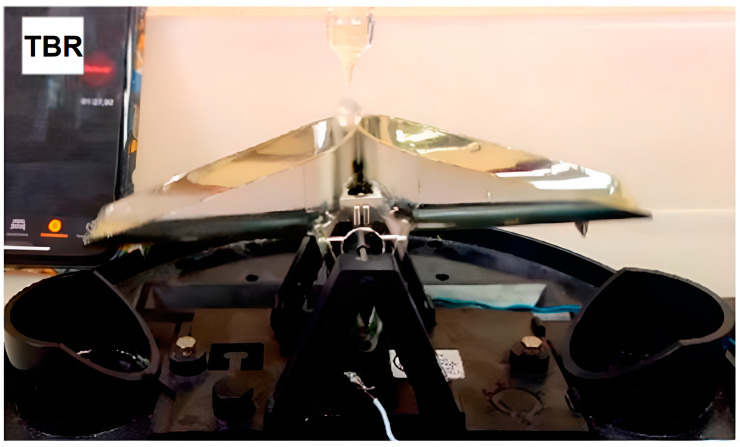
Water falling on the bucket during the tipping movement as an unmeasured surplus. Source: [2].

The mechanical characteristics that benefit the operation of TBRs also generate a systematic mechanical bias that underestimates rainfall as the rainfall intensity (*RI*) increases. According to [63], this phenomenon is due to the water lost during the tipping movement of the buckets. During this movement, some water is not measured since the movement starts at that instant when a given bucket’s volume is reached. However, the bucket tipping rotation requires an amount of time that generates a time window where some rainwater is added as an unmeasured surplus (Figure 2). In addition, according to [2], there is another source of surplus caused by the drop triggering the tipping movement (*D*), with a certain fraction of its volume that exceeds the *Vt*, both related to *RI.* As the *RI* increases, the water flow in the funnel and the size of *D* increases. However, the surplus due to *D* can be considered negligible in contrast to that caused by the time the bucket takes to tip [2,72].

The volume of water lost during the tipping movement is related to the flow rate out of the funnel which, in turn, depends on the *RI*. If a constant increase in the *RI* is assumed, the flow rate would increase as the *RI* does, and therefore, so would the losses. However, they also depend on the tipping time, which decreases as the *RI* increases (especially at a high *RI*), caused by water constantly falling and impacting the rocker arm, accelerating its movement, which also generates a decrease in the *Vt* by reducing the kinetic energy needed to tip. The reduction of these two parameters results in a compensation effect, which offsets part of the losses; therefore, the increase in the volume of water lost during the tipping movement as a function of the increase in the *RI* follows a curved pattern [2,73].

Regarding the effects of the *RI* in TBR measurements, three regions were observed by [2,73]. The first region corresponds to a very low *RI*, where the flow rate falling from the funnel orifice is too low that does not have enough time to fall into the bucket in motion, so no extra water is added during the tipping time. In the second region, the flow rate generated by the *RI* is fast enough to add some water as it drops into the bucket during the tipping time and produces an underestimation in the measurements. The third region is observed at a high *RI* above a threshold where the drip-falling flow becomes a continuous stream and adds not only unmeasured water to the bucket during the tipping time, but also a constant thrust on the rocker arm; this generates a reduction in the *Vt* and tipping time, resulting in compensation of the underestimation effect generated by the unmeasured water falling into the bucket during the tipping time.

The magnitude of the biases depends on the TBR design characteristics, such as the size of the funnel orifice, the size of the funnel, the size of the buckets, and the instrument resolution [2,63,74]. For example, these biases are caused by the time the bucket takes to tip, and they will appear after a certain *RI,* since at a very low *RI*, the funnel flow rate is too low and the time window generated by the tipping time is not long enough to generate a measurement overshoot, which depends on the TBR characteristics. The magnitude of this no tipping time bias or first region varies from one TBR model to another depending in the specific characteristic, as well as the continuous stream threshold [2].

Since the biases are related to the *RI*, calibration strategies as a function of the *RI* are used to reduce their impact on the measurement. Likewise, there are other sources of instrumental error in the buckets’ self-functioning mechanism: drag variations, friction force variation in the fixed axis, variability in *D* size, the interaction of falling droplets with tipping bucket balance, changes in falling water direction, leveled errors, an incomplete evacuation of water from the tipping bucket or funnel, the buckets’ volume variation, water origin, initial conditions of the buckets, wetting loss to the internal wall of the gauging funnel, evaporation, splashing, mechanical blockages, and logging errors. These sources do not depend on the *RI* and calibration cannot avoid them; however they can be reduced by proper equipment management and maintenance [2,23,63,75,76,77,78]. Moreover, a random unexplained variation, not related to the *RI*, has been identified by several authors [2,23,25,71,75,76,79,80,81,82], which can be significant and could be explained by a combination of the sources previously mentioned and other sources originating from environmental agents (e.g., wind) and human error [2,75]. Nevertheless, as [74] affirmed, these random errors are small compared to those derived from *RI* or wind.

### 3.2. External Sources of Bias

**Figure 3:**
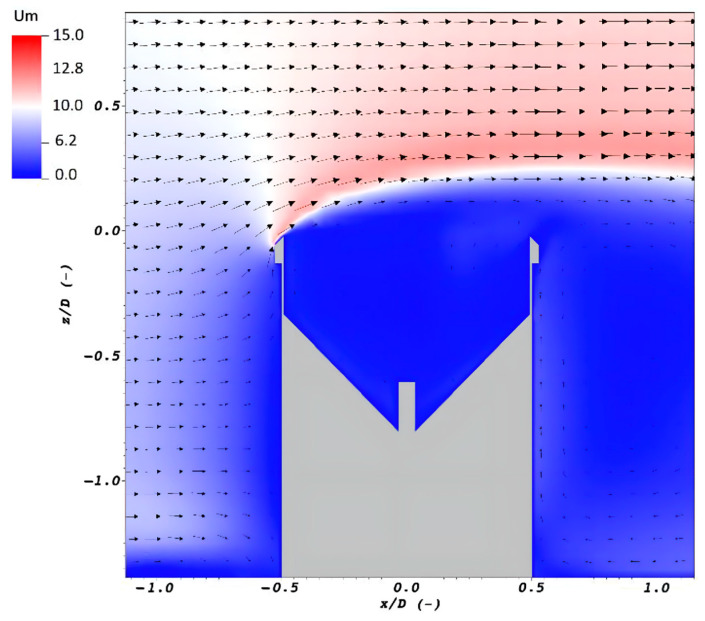
A sample section of the airflow field (velocity magnitude, Um (m s^−1^) along the vertical symmetry of a TBR at a horizontal wind speed of 10 m s^−1^ simulation. The arrows represent the time-averaged magnitude and direction of the airflow. Source: [78,85].

These are the biases caused by external agents, not directly related to the TBR operating mechanism, such as losses from evaporation, wetting, splashing, funnel clogging, colonization for animals or insects, and wind effects [68,71,79,80,82,83,84,85,86]. Among all of these, the last one is the most relevant source of error in many precipitation monitoring sensors. According to [28], the effect of wind is caused by the interaction between the gauge body and the airflow. The rain gauge behaves as a “bluff-body” when impacted by the wind that produces airflow deformations around it, which results in significant acceleration and vertical velocity components above the collector that deviate the trajectories of the hydrometeors (Figure 3) and induces a significant reduction in their performance [28,68,78,85,87]. 

Wind biases can cause approximately 2–10% and 10–50% undercatch for liquid and solid precipitation, respectively. It depends on the gauge geometry, the wind speed, the type of precipitation, and its characteristics such as particle distribution, size, and precipitation intensity [85].

### 3.3. TBR Management and Limitations

TBR biases are generally defined as instrumental and environmental; however, in this paper, we also consider TBR management as an important source of measurement biases, since proper TBR management can either reduce biases or favor their effects. The importance of instrumental management is not usually taken into account during the operation of rainfall monitoring networks or for data users; thus, errors propagate through applications or modeling and the results are affected by limited reliability, since most historic recorded rainfall series have never been corrected and are affected by significant biases and uncertainties [28,65,69,70]. In this section, TBR management error sources and their limitations are presented as spatial sampling errors, solid precipitation measurement bias, data acquisition frequency, and maintenance.

#### 3.3.1. Spatial Sampling Error and Solid Precipitation Measurement Bias

Since TBRs (and rain gauges) estimate point rainfall, one of their major limitations is their capability to capture the spatial variability of rainfall, whose impact on hydrological systems is significant [8,34]. For hydrological management, interpolation diagrams or analysis are commonly used to estimate spatial rainfall variability in rain gauge networks. Moreover, since gauging stations are solely installed in a few locations, dense rain gauge networks do not cover large areas due to the cost of monitoring and maintenance, as well as installation difficulties. Likewise, precipitation can show specific variability (especially in mountainous areas), which affects the reliability of data in hydrological research, i.e., to forecast rainfall or floods based only on rain gauge data, resulting in hydrologists preferring satellite, radar, or merge data to address rainfall spatial variability [6,8,28,88].

Another limitation of TBRs is their low capacity to capture and measure solid precipitation, since the performance of the operating mechanism requires liquid water. Thus, snow accumulates in the funnel, whose storage capacity is not usually large, and measurement is delayed until it thaws. Likewise, in hail conditions, much of the precipitation is lost due to bouncing of the hail in the funnel [89,90,91].

#### 3.3.2. Data Acquisition Frequency

Hydrological research needs real-time hydrological monitoring to explain the nature of dynamical hydrologic systems, ecohydrology, and vegetated flows; this will support decision making regarding flood mitigation risk, improvement in radar *RI* calibration, and reduction of the uncertainty in precipitation measurements, among others [11,12,13,14,16,92,93,94]. The temporal resolution depends on the use of the data and the reliability required. Nevertheless, the tendency should be to obtain data with a high-temporal frequency, so that they can be useful for all users and can lead to better understanding of hydrological processes rather than be conditioned to one single application [93,94].

Rainfall rate estimation and bias corrections are usually performed in minutely, hourly, daily, or monthly totals or even in fixed time intervals for individual precipitation events [31]. A constant *RI* within the chosen sampling time is assumed, where the number of tips is counted and then for *RI* calculation, they are multiplied by the nominal *Vt* and divided by the fixed time interval [65,81]. “This assumption is a simplification of the rainfall continuous phenomenon and leads to the so-called sampling error” [28]. Since the volume of water collected in the bucket is not sufficient to trigger the tip at the set sampling time, this volume is not considered for the measurement, leading to an underestimation during the sampling time and an overestimation once the tip occurs at the next measurement. The same process can be seen in consecutive events, due to the amount of water already stored in the bucket before the start of the event and the water that remains in the bucket at the end of each event [28,65,81].

Sampling errors generate a bias in *RI* estimation and total rainfall duration, which are magnified at a low *RI* due to the time needed to accumulate the *Vt*, since due to the precision of the *TBR*, this bias is attenuated with the increment of *RI* as the higher the *RI*, the shorter the time between the beginning of the event to the first tip. Additionally, it does not allow for rainfall intermittency to be defined, which refers to temporary cessations of rain during a rainfall event, thus increasing the bias in the *RI* estimation [95].

Sampling errors mostly affect the calculation of rainfall rates, as the larger the sampling time, the lower the estimated rainfall rate; thus, sampling errors can be reduced by measuring the rainfall rate with the inter-tip time instead of counting the number of tips within a fixed sampling time [96]. Accordingly to [68], “high-resolution measurements, even down to the scale of the single hydrometeor in some cases, are the way to achieve a better knowledge of the precipitation process and to increase the confidence of users in the accuracy of their basic source of information”. The inter-tip time scale is the best option to improve the performance of TBRs, since it works in the resolution of the instrument [25,28,97].

#### 3.3.3. TBR Maintenance

**Figure 4:**
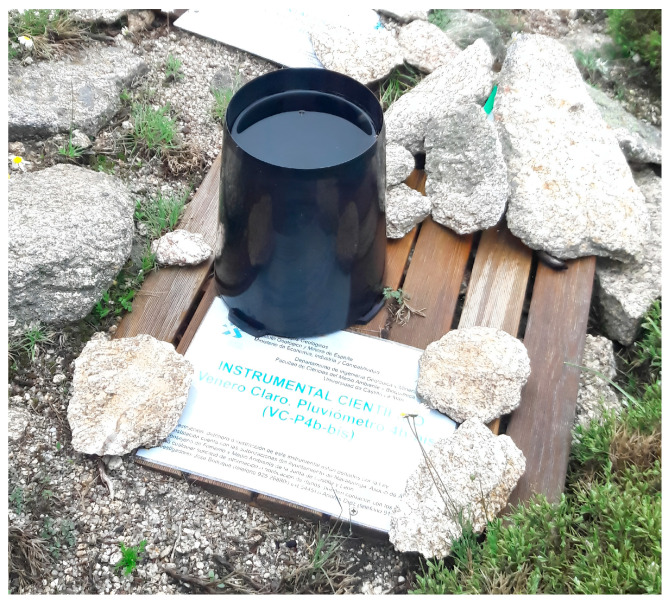
Clogged TBR with rainfall water accumulated in the funnel (IGME, Venero Claro, Spain).

The mechanical operation principle of TBRs (rocker arm, funnel, and moving elements) requires periodical maintenance to guarantee optimal functioning and reduce mechanical and environmental biases to obtain reliable data. Proper installation, cleaning, leveling, and calibration of the equipment is mandatory to guarantee reliability in TBR measurements. The funnel is susceptible to clogging due to the accumulation of dust, tree leaves, and other external elements deposited by the wind or even by the presence of birds that usually perch on them (Figure 4). Dust, small particles, and insects can block the rotating movement of the buckets or modify the balance in the rocker arm [28,31,98].

Exposure to various factors over time produces a variation in the equipment’s operation (unevenness) that may compromise the viability of data. Therefore, maintenance and calibration must be performed frequently; this is not simple to achieve and is often not practiced by the entities in charge of managing monitoring networks, which ends in the magnification of measurement biases [31,64].

## 4. TBR Calibration and Error Reduction Strategies

Calibration is an uncommon practice for the operators of monitoring networks; this could be because of a lack of knowledge or, in many cases, because calibration is time consuming. This paper synthesizes the scientific advances in calibration and error reduction strategies in TBRs. The authors hope that this information will encourage scientific innovation to address the limitations of the most widely implemented rain gauge in the world. Likewise, we hope that the information will be forwarded to rainfall monitoring managers and data users, to improve the reliability of data.

### 4.1. Wind-Induced Bias

In the literature, wind-induced bias is addressed through numerical simulation (computational fluid dynamics (CFD) and particle tracking) and experiments (field and wind tunnel tests) [28,68,85].

#### 4.1.1. Experimental Procedures

Calibration curves are generally derived from experimental data measured via two different precipitation gauges, one under operational conditions, as well a reference one placed in a pit with the gauge orifice at ground level, away from the edge of the pit to avoid internal splashing, as per the World Meteorological Organization’s (WMO) recommendations [64]. The anti-splash grid, with a central opening for the gauge, must cover the pit, except where the gauge orifice is located, according to the standard EN 13798:2010 [99]. The ratio between both gauges’ measurements (gauge under operational conditions (*h_meas_*) and the reference one (*h_ref_*), for a given wind speed (*U)*, is called the collection efficiency (*CE)*, (Equation (1)) [28]. Some examples of adjustment curves derived from field measurements can be found in the literature [100,101].

(1) $$CE=\frac{h_{meas}(U)}{h_{ref}}$$

#### 4.1.2. Numerical Simulation

Adjustment curves derived from field experiments are strictly dependent on the site where the test was located, the associated precipitation, wind, and the reliability of the reference gauge. In contrast, theoretical approaches allow for a complete coverage of various local characteristics to be achieved; the increase in processing capacity literature has focused on a physical numerical approach where adjustment curves are derived as a function of precipitation intensity and wind speed [28]. Exploiting the potential of numerical resolution for the basic equations of fluid motion and particle–fluid interactions allows the airflow patterns (acceleration, velocity, and turbulence) produced by the aerodynamic response of the gauge geometry to be established [79,87,102,103].

Particle tracking and CFD simulation can be performed by different models such as the Unsteady Reynolds Average Navier Stokes (URANS) or the Reynolds Average Navier Stokes (RANS) equations, Lagrangian particle tracking (LPT), and large eddy simulation (LES). Some examples of wind bias numerical simulations can be found in the literature [28,78,85,87,103].

Numerical approaches based on CFD and LPT simulations are usually validated by comparing them with wind tunnel measurements and allow thee adjustment curves for wind-induced bias to be derived by simulating different gauge shapes, precipitation intensity, and wind speed combinations. The *RI* is best correlated with wind-induced biases [78,85,100].

### 4.2. TBR Instrumental Calibration

The calibration of TBRs is often needed to reduce instrumental measurement uncertainty. Traditional calibration methods are complex, time consuming, and are often not implemented by monitoring network operators due to a lack of knowledge and a lack of manufacturer recommendations. Several effective calibration methods have been proposed over the years: methods based on rain gauge parameters and analytical procedures [2,63,73,75,104,105,106], laboratory determination of calibration curves [72,77,96,107], comparison with other types of rain gauges, etc. [25,82,108]. However, calibration procedures can be classified into static and dynamic calibrations.

#### 4.2.1. Static Calibration

This calibration is based on leveling the TBR and adjusting the adjustable stop crew, under the bucket, and then adding water drop by drop with a nozzle until a given water volume tips the bucket. This procedure is repeated several times until the tipping volume for each bucket fixes the volume tip expected. It is commonly recommended by manufacturers for calibration purposes, to ensure a fixed *Vt* in both buckets, and it assumes a constant water volume for tipping the bucket regardless of the *RI*, which is why it is called static calibration. Despite being the fastest and easiest calibration method, it is also a necessary step in other calibration procedures and should always be performed, even when manufacturers affirm that the TBR comes factory calibrated. Nonetheless, it does not consider biases related to the *RI* variation, water source (rainwater, tap water, etc.), or the initial bucket conditions, causing important variations in the *Vt* as described in Section 3 [2,72].

#### 4.2.2. Dynamic Calibration

Dynamic calibration is based on calibrating the TBR while the bucket is in motion. It compares the measured rain gauge rates with actual rainfall rates, which are calculated from applied flow rates by using a precision pump and a high-resolution weighing device, to derive a calibration curve and catch the systematic mechanical bias that underestimates rainfall as the *RI* increases [28,72].

Catching gauge calibration and dynamic calibration in TBRs have been internationally standardized by the EN 17277:2019 Standard [109], which deals with instrument calibration measurement requirements and the classification of rainfall instruments. On the contrary, non-catching-type gauges do not have standards for calibration [68].

According to EN 17277:2019, tests at different *RIs* must be performed in a certified laboratory with a given and constant flow rate (verified by weighing) for a period of at least 30 min per test, where the sampling time for RI calculation should be 1 min. Then, a dynamic calibration curve can be determined either theoretically or in the form of a best-fit regression function. Static calibration is required as a previous step to ensure an accurate balance of the two buckets and the nominal *Vt*. A deviation of ±5% is acceptable.

EN 17277:2019 provides a classification based on the performance obtained in the calibration test results, where three performance classes are indicated: Classes A, B, and C (Table 1). The deviation should be calculated by Equation (2).

(2) $$e_{real}=\frac{I_{meas}-I_{ref}}{I_{ref}}\times 100$$

where *e_real_* is the percentage relative deviation, *I_meas_* is the measured *RI*, and is *I_ref_* the reference *RI*.

#### 4.2.3. TBR Field Calibration and Maintenance

TBR maintenance and checks in the field should be performed at regular intervals, including the cleaning of dirt and debris that has accumulated in the funnel and buckets, checking the levelness of the equipment, verifying time interval dates and records, and also checking on-site calibrations to detect possible malfunctions of the gauge and any deterioration of the instrument’s performance [64,68,98]. The WMO [64] has developed and recommended a procedure (an abbreviated laboratory calibration, to use a lower volume of water) for measuring the reference intensity in the field using a portable calibration device [28], capable of generating a constant water flow for any single test for at least three different flow rates.

### 4.3. TBR Design Modification

As seen in the previous sections, some TBR biases are strongly influenced by the TBR’s characteristics, so modification of the designs can reduce the effect of biases in precipitation measurements. Some attempts have been performed by modifying the bucket profile, such as the use of a siphon tube to deliver a pre-set volume of collected water to each bucket or self-powered wireless TBR, changing the shape of the instrument with the addition of components such as combining tipping and weighing in the measurement. However, they have not avoided the errors even under optimum conditions, but have just minimized them [16,64,110,111]. It is common to find cylindrical TBRs; however, different shapes have recently been developed to reduce wind biases such as triangular and calix-shaped external cases [28,64,85,87].

The design, size, shape, funnel orifice, material, and other design elements have a strong influence in TBR biases; for example, just by changing the falling distance of the bucket, the magnitude of the biases can be reduced or increased, as was shown by [16], so a proper design of the instrument to optimize the parameters can reduce biases in rainfall measurement.

### 4.4. Spatial–Temporal Sampling Error Reduction Strategies

As was commented on in the preceding sections, TBRs are single-point sensors and their measurements are hardly representative of the variability (space and time) that characterizes the precipitation over large areas. Nonetheless, most hydrological processes respond to the spatial distribution of rainfall (space and time) over the surface, and their understanding and quantification largely rely on the knowledge of the spatial variability of rainfall. In hydrological research, it is mandatory to collect accurate data with a high spatial–temporal frequency to better understand hydrological systems. The appropriate location, traceability, and number of TBRs of a rainfall monitoring network are fundamental indicators of their overall quality and accuracy [2,6,28].

According to [28], the focus in terms of defining an optimal location, type of gauge, and gauge density should be on the accuracy and traceability of measurements to increase the reliability of monitoring networks, instead of on the purpose of the measurement (e.g., modeling, flood forecast, or irrigation) and the morphological characteristics of the region where the network is deployed (e.g., accessibility, vegetation coverage, or slope). Of course, these parameters are also important; however, the accuracy and reliability of data should be the main factors under consideration, not defining accuracy in terms of purpose, limiting the potential uses of the data.

Recommendations for the minimum densities of WMO precipitation monitoring stations can be seen in [98]. Other methods proposed in the literature for optimizing the density and location of rain gauge stations are summarized in [112]. These include aspects such as economically efficient techniques, clustering techniques, least square methods and entropy, covariance and variance analyses, kriging, information entropy, geographic information system (GIS)-combined kriging, and information entropy.

TBR radar or TBR remote sensing monitoring networks are other alternatives to capture the spatial variability of rainfall, to improve the short-term predictability synergizing the capabilities of both systems to obtain more reliable data. Thus, they can improve radar or remote sensing measurements. However, the accuracy of radar measurements is generally insufficient, particularly in the case of extreme rainfall magnitudes, as described in Section 2 [4,94].

In the case of temporal sampling errors that mostly affect the calculation of rainfall rates and the duration of rainfall events, the larger the sampling time, the lower the estimated rainfall rate; thus, sampling errors can be reduced by measuring the rainfall rate with the inter-tip time instead of counting the number of tips within a fixed sampling time [96]. According to [68], “high-resolution measurements, even down to the scale of the single hydrometeor in some cases, are the way to achieve a better knowledge of the precipitation process and to increase the confidence of users in the accuracy of their basic source of information”. The inter-tip time scale is the best option to improve the performance of TBRs, since it works in the resolution of the instrument [25,28,97]. However, it will not eliminate the biases in the *RI*, especially at a low *RI*, to improve the *RI* estimation; as [95] demonstrated, an acoustic recording apparatus can be set near the TBR to define rainfall behavior and calibrate the *RI* in the inter-tip time.

## 5. Discussion and Future Perspectives

In the future, it is foreseen that TBRs will continue to be one of the most widely used pieces of equipment for rainfall monitoring due to their accuracy, low cost, simplicity, and low-energy consumption compared to other technologies [2]. Moreover, new technological advances and more widespread estimation methods such as remote sensing or radars require this type of ground sensor for the calibration and validation of their measurements to increase their spatial–temporal resolution and accuracy [28,59]. Thus, many works have focused and will continue to focus on their main disadvantage: measurement biases (mainly those caused by wind and mechanical biases) [16,68,75,84,85,87,106].

Although many of these measurement biases can be improved by modification in terms of the design of the equipment, only a few advances have been made in recent years, which have focused on the modification of the shape of rain gauges (to reduce the effects of wind) [85], the modification of certain design features, and the implementation of technologies for energy self-supply [111]. Likewise, the efforts of the scientific community have focused either on calibration processes to obtain more reliable measurements, with a growing interest in obtaining a higher amount of data with high temporal and spatial frequencies to better understand the nature of hydrological phenomena [16], or focusing on calibration procedures that can be applied in real time or at an inter-tip time [28,97], to avoid spatial–temporal sampling error, which is an important limitation in TBR measurements.

In our understanding, spatial–temporal sampling error in TBRs is a major issue to overcome to improve the accuracy of the data obtained using TBRs to catch the variability of rainfall not only in space, but also in time. Many efforts have been performed to improve the TBR location among basins [2,6,28,98,112], responding to different criteria, and to reduce the monitoring cost to increase the number of TBRs in the field [16]. Nowadays, merged TBR radar or remote sensing technologies are becoming an interesting alternative to catch spatial rainfall variability among a basin, combining the advantages of these technologies to achieve better results [4,94].

Spatial–temporal sampling errors are the major limitation of TBR equipment and are related directly to the instrument. From our point of view, the best alternative to overcome these limitations is a combination of TBRs with other sensors; for example, *RI* measurements using TBRs can be improved up to an inter-tip time [96], but at a low RI, biases will continue to be significative, so a combination with other sensors such as acoustic recording apparatus can be the solution to overcome these limitations [95]. If we want to overcome spatial–temporal sampling errors in rainfall monitoring, we need to think about the use of more than one specific sensor, since merged solutions seem to be the best alternative to synergize the advantages of the different monitoring technologies and overcome their limitations.

On the contrary, regarding the efforts to reduce mechanical and external source induced biases, studies have mostly centered on the wind and instrumental losses related to the *RI* during the tipping time (the most significative source of biases). These studies have sought to develop methodologies and algorithms to correct errors in real time, focusing on numerical simulations and physical–mathematical principles beyond current experimental procedures; nevertheless, they also need experimental procedures to be implemented [2,28,75,80,84,87,105,106]. New field and laboratory calibration equipment and methods have also been developed to facilitate calibration processes and reduce operation time [106], especially for dynamic calibration, which was standardized a few years ago in EN 17277:2019. However, we think corrections need to be automatically performed by algorithms to simplify the correction and calibration processes for operators and data users.

In this work, we cited different procedures, methods, and algorithms developed over the years [2,25,63,73,74,76,78,83,97,103,104,105,106,107]; however, we focused on the two main calibration procedures: static calibration, a basic calibration procedure that must be performed as a prior step in other calibration procedures to guarantee the *Vt*, and dynamic calibration, which has been standardized and has proven to be an effective calibration method (EN 17277:2019).

Proper TBR maintenance must be employed to guarantee optimal functioning and reduce mechanical and environmental biases to obtain reliable data. Installation, cleaning, leveling, and frequent field calibration of the equipment should be mandatory [28,31,98]. Therefore, it is not simple to achieve and is often not practiced by the entities in charge of monitoring networks, which ends in an increase in measurement biases [31,64].

Despite the efforts made by the scientific community to develop new calibration procedures and design modifications, this problem has not been overcome; biases cannot be completely eliminated, but their effect can be significantly reduced. However, there is a gap in the dissemination and use of these procedures by data users and those in charge of handling TBRs. Therefore, while new procedures are developed to overcome biases in rainfall measurement, in the near future, efforts for their dissemination, among data users and network operators who are responsible for the maintenance of the calibration of the equipment and therefore for the reliability of the data, must be made. Thus, all of this effort can achieve better results, obtaining a more reliable database to better understand hydrological phenomena and achieve best-fit simulations and forecasting for hydrological management, research, and decision making.

## 6. Conclusions

The need for precipitation data has increased in recent years, as has the interest in hydrological research and forecasting due to water scarcity and extreme events that cause damage to human activities. The interest in understanding hydrological phenomena produces a high demand for data (space and time), especially reliable data for producing reliable results. This objective will not be achieved without working on the accurate calibration of measuring equipment, an aspect often neglected by hydrological network managers and data users, which propagates the bias in databases and data applications, and consequently, most of the hydrological research performed with this data in pass years is biased.

To overcome spatial–temporal sampling errors (the major limitation of TBRs), merged solutions with other sensors or rainfall monitoring technologies must be considered, since TBRs are limited by proper TBR characteristics and precision. Therefore, the best way to accurately overcome this is through combination with other sensors, such as acoustic recording sensors for temporal measurements and extended radar or remote sensing technologies, to cover these limitations and synergize them.

Calibration methods are time consuming and difficult to implement, but they can drastically reduce TBR measurement biases, although such biases cannot be completely eliminated, even under the current status of technological development and scientific knowledge. Moreover, most rainfall monitoring network operators and data users are unaware of this issue, and most TBR data obtained worldwide do not consider calibration. Therefore, new procedures and equipment to facilitate and reduce the time needed for calibration must be developed. Calibration strategies with faster performance and easier automatic implementation are needed, to reduce the role of operators and data users in calibration. Additionally, proper dissemination of these issues will help to ensure that the knowledge reaches the different actors in charge of the monitoring, processing, logging, and application of the data.

In the above context, this work summarized the equipment and methodologies used worldwide for precipitation measurement, as well as the main measurement biases, uncertainties in calibration, and error reduction strategies in TBRs. We provided a brief review of the knowledge developed in the area to make it more accessible to researchers but also to those in charge of monitoring networks and data users.

## Figures and Tables

**Table 1:** Rain gauge classification ^1^.

Class	Maximum Deviation	Temporal Resolution
A	±3%	1 min
B	±5%	1 min
C	±10%	1 min
Cannot be classified	>±10%	1 min

^1^ Elaborated based on EN 17277:2019.

## Data Availability

Not applicable.
